# Comparative Effectiveness of 2 Diabetes Prevention Lifestyle Programs in the Workplace: The City and County of San Francisco Diabetes Prevention Trial

**DOI:** 10.5888/pcd17.190396

**Published:** 2020-05-28

**Authors:** Assiamira Ferrara, Julia C. McDonald, Susan D. Brown, Janet G. Alexander, Jennifer L. Christian-Herman, Stephanie Fisher, Charles P. Quesenberry

**Affiliations:** 1 Division of Research, Kaiser Permanente Northern California, Oakland, California; 2 Kaiser Foundation Health Plan, Oakland, California; 3 San Francisco Health Service System, San Francisco, California

## Abstract

**Introduction:**

Data on the comparative effectiveness of Diabetes Prevention Programs (DPPs) in the workplace are limited.

**Methods:**

Between September 2015 and July 2016, employees of the City and County of San Francisco who were at risk for type 2 diabetes (N = 158) were randomly assigned to one of 2 DPP-derived programs recognized by the Centers for Disease Control and Prevention: an in-person YMCA-DPP (n = 78) or an online virtual lifestyle management DPP (VLM-DPP) offered through Canary Health (n = 80). The primary outcome was change in body weight assessed at 6 and 12 months. Follow-up ended in August 2017.

**Results:**

Both the YMCA-DPP and VLM-DPP yielded a significant reduction in percentage body weight at 6 months. For the YMCA-DPP, mean percentage change at 6 months was −2.70% (95% confidence interval [CI], −3.91% to −1.48%) and at 12 months was −2.46% (95% CI, −4.24% to −0.68%). For the VLM-DPP, mean percentage change at 6 months was −2.41% (95% CI, −4.07% to −0.77%) and at 12 months was −1.59% (95% CI, −3.51% to 0.33%). The mean between-condition difference at 6 months was −0.25% (95% CI, −2.04% to 1.55%) and at 12 months was −0.84% (95% CI, −3.03% to 1.34%). No significant differences were observed between conditions.

The YMCA-DPP had a slightly higher reduction in waist circumference than VLM-DDP at 6 months (mean between-condition difference −2.00 cm [95% CI, −4.24 to 0.25 cm]). Participant engagement, expressed as mean number of completed core program sessions, was significantly higher for the YMCA-DPP than the VLM-DPP. Participants of the YMCA-DPP completed an average of 10.2 sessions (95% CI, 9.0 to 11.4), and participants of the VLM-DPP completed an average of 5.9 sessions (95% CI, 4.7 to 7.1). The adjusted mean between-condition difference was 4.2 sessions (95% CI, 2.54 to 5.99).

**Conclusion:**

Both the YMCA-DPP and VLM-DPP yielded weight loss at 6 months, which was maintained at 12 months in the YMCA-DPP. The workplace may be an effective setting to offer DPPs.

**Summary**

**What is already known on this topic?**

To our knowledge, this is among the first randomized controlled trials to evaluate lifestyle programs recognized by the Centers for Disease Control and Prevention’s Diabetes Prevention Recognition Program, implemented in the workplace, and delivered by trained staff from the recognized organizations.

**What is added by this report?**

In this trial, 158 City and County of San Francisco employees at high risk for type 2 diabetes were randomly assigned to an in-person YMCA program or an online virtual lifestyle management program to assess the effectiveness of both programs on weight loss among its participants.

**What are the implications for public health practice?**

Weight loss of participants of both programs was significant at 6-month follow-up, but the amount lost did not differ significantly between the 2 programs. We found that the workplace may be an effective venue in which to offer Diabetes Prevention Programs.

## Introduction

An estimated 84 million people in the United States have prediabetes (1,2). The Diabetes Prevention Program (DPP) demonstrated that a lifestyle intervention can reduce the incidence of type 2 diabetes by 58% in high-risk adults (3). DPP-derived lifestyle interventions thus present an opportunity to improve health and reduce the annual cost of type 2 diabetes, which is estimated at $176 billion in direct medical expenses and $69 billion in indirect costs related to absenteeism, lost productivity, and disability (4).

The Centers for Diseases Control and Prevention (CDC) developed the National DPP (5) with the goal of implementing a standardized, evidence-based DPP lifestyle program. CDC established the Diabetes Prevention Recognition Program (DPRP) (6) to monitor and support the delivery of the National DPP. CDC offers recognition to organizations delivering a DPP-derived intervention if they use a CDC-approved curriculum and meet intensity and duration requirements (7).

Given the availability of DPP-derived lifestyle interventions and the high costs of diabetes, employers are increasingly considering offering type 2 diabetes prevention programs to their at-risk employees. Because of its convenience, the workplace holds promise for reaching adults who have barriers (eg, time, transportation), including individuals with fewer socioeconomic resources, that may prevent them from engaging in DPPs (8–10). However, data on the comparative effectiveness of DPRP-recognized lifestyle programs implemented in workplace settings are limited (9,10).

Researchers at the Kaiser Permanente Northern California Division of Research collaborated with the City and County of San Francisco (CCSF) Health Service System to conduct a randomized controlled trial comparing the effectiveness on weight loss of 2 DPRP-recognized lifestyle programs implemented in the workplace setting: 1) YMCA-DPP (11), an in-person program delivered by YMCA-trained staff; and 2) Virtual Lifestyle Management DPP (VLM-DPP) (12), offered through Canary Health, which uses an online platform with supplemental secure email messaging with VLM-trained staff.

## Methods

This study was approved by the Kaiser Permanente Northern California institutional review board. All participants provided written informed consent.

### Study design

The CCSF Diabetes Prevention Trial was a 2-arm, parallel, randomized controlled trial to compare the effectiveness of the YMCA-DPP and VLM-DPP in 7 worksites of the CCSF. Study staff enrolled CCSF employees from each worksite location in waves. At each worksite, enrollment lasted approximately 1 month. Participants were enrolled in September 2015 at the first worksite and in July 2016 at the seventh worksite. Follow-up ended in August 2017.

Participants were CCSF employees. Department contacts from the 7 participating locations promoted the study using flyers, department-wide emails, announcements at meetings, and information on the CCSF website. Interested CCSF employees attended an onsite open information session where research staff explained the 2 DPP interventions; described what participation in the research study would entail; conducted eligibility screening; and assessed height, weight, and waist circumference. Employees were considered eligible based on results from a CDC prediabetes screener (13), which defines high risk as a score of 9 or higher and having a body mass index (BMI, determined by assessing weight in kg divided by height in m^2^) of ≥25.0 kg/m^2^ or ≥23 kg/m^2^ if Asian. Criteria for exclusion were having a major health condition (ie, self-reported diabetes, heart disease, renal insufficiency, cancer, or high blood pressure), not having regular access to a computer or the internet, current enrollment in another weight loss program, or plans to move or change departments in the next 12 months.

Eligible employees were asked to attend an orientation session 1 week later, during which research staff further explained the study. Employees who were interested in participating provided written informed consent and completed surveys.

The study project manager randomly assigned employees to either the in-person YMCA-DPP delivered at the worksite or the online VLM-DPP. An adaptive randomization procedure (14) was used to ensure that the 2 groups remained balanced overall and in terms of the following key characteristics: age (<50 y or ≥50 y), BMI (<30 kg/m^2^ or ≥30 kg/m^2^), sex (male or female), race/ethnicity (white, African American, Hispanic, Asian, or multiracial/other), and job type (office or not office [ie, public works, transportation, or other field-based job]).

### DPP-derived lifestyle interventions

All participants were given the goal of losing 5%–7% of their body weight through dietary change and participation in 150 minutes of moderate-intensity physical activity per week.

The YMCA-DPP, an in-person intervention of 16 weekly, 1-hour group sessions (core curriculum) and 3 biweekly and 5 monthly group sessions (maintenance curriculum) (11), was held for groups of 8 to 15 employees at each participating CCSF worksite at a time convenient for employees (ie, during breaks or lunch). Group sessions were facilitated by trained YMCA-DPP lifestyle coaches and covered the key topics of healthy eating, increasing physical activity, reducing stress, problem solving, and motivation to maintain behavior changes. Participants weighed in with the lifestyle coach at the beginning of each hour-long session and were asked to self-report their minutes of physical activity. They were also asked to self-monitor their food intake and activity daily, beginning from session 1 of the program.

Participants assigned to the VLM-DPP, an online program offered through Canary Health (12), first received a single in-person group orientation where they received instructions on how to use the online platform. The VLM-DPP curriculum included 16 weekly educational sessions (core curriculum) and 8 monthly maintenance sessions to be completed online at a convenient time and place for the participant. Sessions lasted from 25 to 45 minutes and included streaming audio and interactive visual material. Trained VLM-DPP coaches engaged participants using secure messaging to discuss progress, encourage regular participation, and respond to participants’ comments and questions. The program included behavior change techniques such as goal setting and self-monitoring of diet, physical activity, and weight using the online platform.

### Assessments

Assessments were conducted by trained research staff blinded to intervention assignment at baseline, 6 months, and 12 months. Data on demographic characteristics and information on whether a participant’s job was office-based or not office-based (such as public works, transportation, or other field-based jobs) were collected at baseline. Height was measured at baseline using a Seca stadiometer (Seca GmbH). Weight and waist circumference were measured at baseline and at 6 months and 12 months. Weight was measured using a Tanita scale (Tanita Corporation). All body measurements were taken according to standard protocols (15) with participants in light clothing and without shoes, with measurements taken in duplicate to the nearest 0.1 pound or 0.1 centimeter. The average of the 2 measurements was used if the difference between them was less than 1.0 pound or 1.0 centimeter; otherwise, a third measurement was taken and used. Weight and height measured at baseline were used to calculate BMI. Waist circumference was measured by positioning a tape measure 1 inch above the umbilicus at the end of the participant’s normal expiration. The mean value of waist circumference was calculated if the 2 initial measurements agreed within 1 centimeter. Otherwise, an additional measurement was taken, and the third recording was used.

Diet and physical activity were assessed at baseline and the 6-month and 12-month follow up. The Block Fat Screener was used to assess diet (16), and the International Physical Activity Questionnaire (17) was used to assess physical activity. The number of intervention sessions completed between random assignment and 6 and 12 months was assessed by YMCA group facilitators and the VLM online platform.

### Outcomes

The primary outcome was body weight expressed as change in the amount of body weight in kilograms and percentage change in body weight. Secondary outcomes included changes in waist circumference; physical activity, expressed as duration and intensity per week; dietary fat intake; and participant engagement, expressed as mean number of intervention sessions completed and the proportion of participants who completed 4 or more sessions.

### Power and statistical analyses

The study sample of 80 individuals per intervention condition provided 80% power to detect as small as a 1.8% difference in mean percentage weight loss between intervention conditions, assuming a standard deviation for percentage weight loss of 4% (9,10). In addition, with 80 individuals in each condition, the minimum detectable difference in mean weight change was 2.2 kg, assuming a standard deviation for weight change of 5 kg (9,10).

Linear mixed effects regression techniques were used to model change in weight, percentage change in body weight, waist circumference, physical activity, and dietary fat intake, as a function of intervention condition and time, controlling for baseline variables used in the adaptive randomization procedure (age, BMI, sex, race/ethnicity, and job type) (18). Additional models included an intervention condition-by-time interaction term, which enabled us to assess the variation in group differences in means over time. Model parameters were estimated via maximum likelihood methods. Modified Poisson regression (19) for estimation of relative risk (RR; ratio of proportions) was used to compare conditions on the proportion attending 4 or more core sessions, and linear regression analysis was used to compare conditions on the mean number of completed core sessions. Two sets of analyses were conducted for weight and waist circumference outcomes: 1) an analysis including only individuals with clinic-measured weights and waist circumferences, and 2) an analysis including all randomized individuals by using measured or imputed weights and waist circumferences and if measurements were missing, using multiple imputation techniques. The chained equation technique (20) was used to generate 20 imputed data sets, with discriminant function and multiple linear regression analysis used as imputation models for missing categorical and continuous covariates, respectively. Statistical analyses were performed on each of the imputed data sets, with results combined using Rubin’s rules (21) providing valid point and interval estimates appropriately accounting for the uncertainty in imputing the missing data (21). The analyses for diet and physical activity outcomes included only individuals who completed the related questionnaires. All analyses were conducted using SAS version 9.3 (SAS Institute, Inc). 

## Results

A total of 351 CCSF employees attended the information session at their worksite, and 158 (45%) were randomly assigned to either the YMCA-DPP (n = 78) or the VLM-DPP (n = 80) (Figure). Employees who were randomly assigned were significantly more likely to be female, have a postgraduate degree, and have an office-based job compared with those who were not randomly assigned (*P* < .05). Baseline characteristics of randomly assigned participants did not differ significantly by intervention condition (Table 1). For follow-up assessments, 116 (73%) participants had at least 1 clinic visit and measured weight during the 12-month follow-up. Weight measured during follow-up was available for 65 (83%) of those assigned to the YMCA-DPP and 51 (64%) of those assigned to VLM-DPP (Figure). Participants who completed 1 or more lifestyle program sessions were significantly more likely to have a postgraduate degree and an office-based job compared with those who did not engage (*P* < .05). Intervention engagement differed by condition; 71 (91%) participants in the YMCA-DPP and 59 (74%) participants in the VLM-DPP completed 1 or more sessions (*P* = .005).

**Figure:**
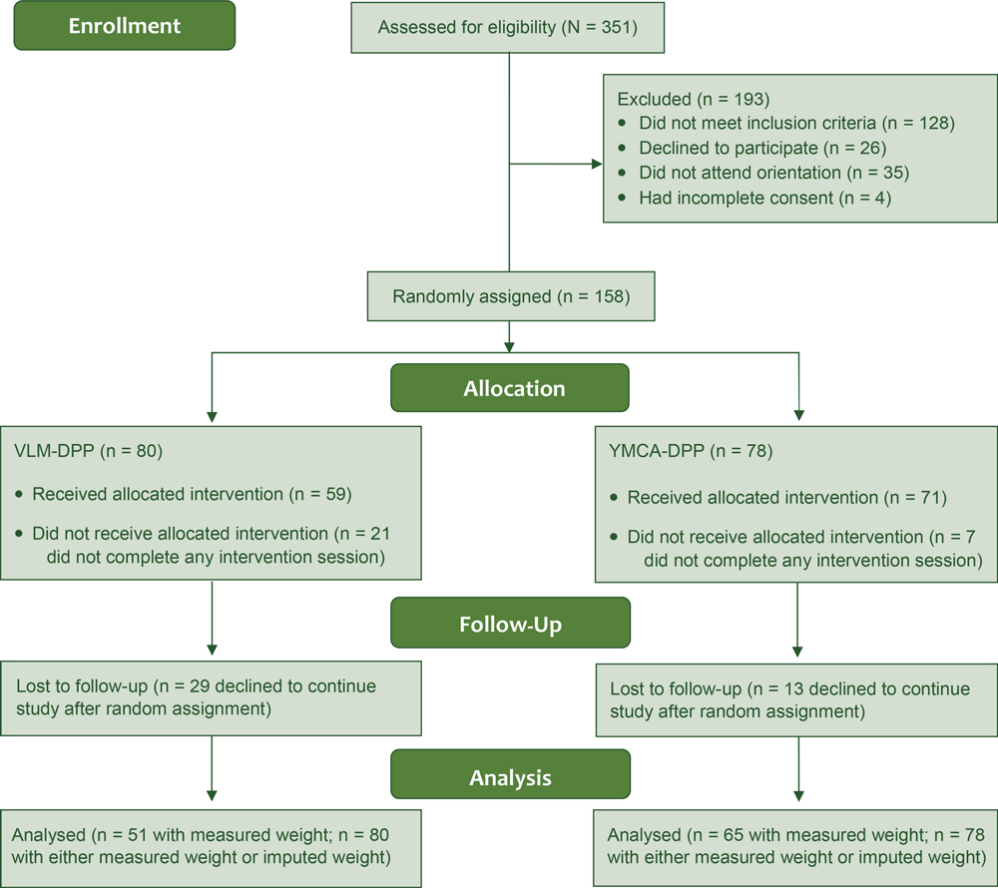
Random assignment into 1 of 2 diabetes lifestyle management programs in the workplace, the City and County of San Francisco Diabetes Prevention Trial, 2015–2016. Abbreviations: DPP, diabetes prevention program; VLM, virtual lifestyle management.

**Table 1:** Baseline Characteristics of Employee Participants, by Intervention Condition, the City and County of San Francisco Diabetes Prevention Trial, 2015–2016^a^

Characteristic	YMCA DPP (N = 78)	Virtual Lifestyle Management DPP (N = 80)
**Female sex**	56 (71.8)	59 (73.8)
**Age, y**		
30–39	8 (10.26)	8 (10.26)
40–49	17 (21.79)	20 (25.64)
50–59	32 (41.03)	37 (47.44)
≥60	21 (26.92)	13 (16.67)
**Race/ethnicity**		
African American	12 (15.4)	13 (16.3)
Asian	24 (30.8)	24 (30.0)
Hispanic/Latino	11 (14.1)	11 (13.8)
Other/mixed	11 (14.1)	11 (13.8)
White	20 (25.6)	19 (23.8)
Missing	0	2 (2.5)
**BMI, kg/m^2^**		
≤24.9	7 (9.0)	5 (6.3)
25.0–29.9	27 (34.6)	28 (35.0)
30.0–34.9	20 (25.6)	28 (35.0)
≥35.0	24 (30.8)	19 (23.8)
**Job type**		
Office	67 (85.9)	68 (85.0)
Nonoffice	11 (14.1)	12 (15.0)
**Education**		
High school graduate or less	4 (5.1)	4 (5.0)
Technical/trade school, some college	23 (29.5)	17 (21.3)
4-Year college graduate	29 (37.2)	28 (35.0)
Postgraduate degree	21 (26.9)	28 (35.0)
Missing	1 (1.3)	3 (3.8)
**Marital status**		
Married	43 (55.1)	47 (58.8)
Not married, living with partner	4 (5.1)	3 (3.8)
Divorced/separated	16 (20.5)	10 (12.5)
Single (never married)	12 (15.4)	14 (17.5)
Widowed	3 (3.8)	4 (5.0)
Missing	0	2 (2.5)
**Mean (SD) age, y**	52.7 (8.7)	51.8 (7.8)
**Mean (SD) BMI, kg/m^2^**	32.1 (5.9)	31.8 (5.5)
**Mean (SD) weight, kg**	88.5 (22.5)	87.7 (20.3)
**Mean (SD) waist circumference, cm**	105.3 (15.2)	104.6 (14.0)

Abbreviations: BMI, body mass index; DPP, Diabetes Prevention Program; SD, standard deviation.

a Values are no. (%) unless otherwise indicated. All *P* values for condition differences ≥.05.

In the analysis using multiple imputed measurements, during the follow-up period, participants in the YMCA-DPP lost more weight on average than those in the VLM-DPP; however, this difference was not significant, with an average adjusted mean between-condition difference of −0.21 kg (95% confidence interval [CI], −1.74 to 1.32 kg) (Table 2). Both the YMCA-DPP and VLM-DPP yielded a significant reduction in weight at 6 months. Mean weight change for the YMCA-DPP was −2.14 kg (95% CI, −3.21 to −1.07 kg) and for the VLM-DPP was −1.97 kg (95% CI, −3.33 to −0.60 kg); the mean between-condition difference was −0.14 kg (95% CI, −1.67 to 1.39 kg). Weight loss remained significant at 12 months in the YMCA-DPP, but not in the VLM-DPP. The mean weight change in the YMCA-DPP was −2.04 kg (95% CI, −3.63 to −0.45 kg) and in the VLM-DPP was −1.27 kg (95% CI, −2.93 to 0.39 kg); the mean between-condition difference was −0.74 kg (95% CI, −2.63 to 1.15 kg). On average during the follow-up period, we found no difference between conditions in percentage change in body weight (average adjusted mean condition difference, −0.34% [95% CI, −2.12% to 1.44%]). Mean changes in percentage body weight at 6 months were −2.70% (95% CI, −3.91% to −1.48%) for the YMCA-DPP and −2.41% (95% CI, −4.07% to −0.77%) for the VLM-DPP (mean condition difference, −0.25% [95% CI, −2.04% to 1.55%]). Mean changes in percentage body weight at 12 months were −2.46% (95% CI, −4.24% to −0.68%) for the YMCA-DPP and −1.59% (95% CI, −3.51% to 0.33%) for the VLM-DPP (mean between-condition difference, −0.84% [95% CI, −3.03% to 1.34%]). Compared with the VLM-DPP, the YMCA-DPP on average had a greater reduction in waist circumference at 6 months, although it was not significant (average adjusted mean between-condition difference, −2.00 cm [95% CI, −4.24 cm to 0.25 cm]) (Table 2). The analysis that included only individuals with measured weights or waist circumferences yielded similar results (Table 2).

**Table 2:** Mean Changes in Body Weight and Waist Circumference From Baseline to 6-Month and 12-Month Follow-Up, With Mean Differences Between Intervention Conditions, City and County of San Francisco Diabetes Prevention Trial, 2015–2016

Characteristic	Measured Body Weights or Waist Circumferences						Measured or Imputed Body Weights or Waist Circumferences					
	YMCA-DPP		VLM DPP		Adjusted^a^ Mean Condition Difference (95% CI)	*P*	YMCA-DPP		VLM DPP		Adjusted^a^ Mean Condition Difference (95% CI)	*P*
	N	Mean (95% CI)	N	Mean (95% CI)			N	Mean (95% CI)	N	Mean (95% CI)		
**Body weight, kg**												
Average effect of intervention	78	—	80	—	−0.58 (−1.93 to 0.76)	.39	78	—	80	—	−0.21 (−1.74 to 1.32)	.79
6 months	63	−2.20 (−3.07 to −1.33)	48	−1.83 (−2.92 to −0.75)	−0.52 (−1.87 to 0.82)	.44	78	−2.14 (−3.21 to −1.07)	80	−1.97 (−3.33 to −0.60)	−0.14 (−1.67 to 1.39)	.86
12 months	54	−2.29 (−3.66 to −0.92)	43	−1.44 (−2.49 to −0.39)	−1.00 (−2.61 to 0.61)	.22	78	−2.04 (−3.63 to −0.45)	80	−1.27 (−2.93 to 0.39)	−0.74 (−2.63 to 1.15)	.44
**Percentage body weight**												
Average effect of intervention	78	—	80	—	−0.80 (−2.33 to 0.73)	.30	78	—	80	—	−0.34 (−2.12 to 1.44)	.71
6 months	63	−2.81 (−3.81 to −1.81)	48	−2.32 (−3.48 to −0.98)	−0.73 (−2.26 to 0.81)	.35	78	−2.7 (−3.91 to −1.48)	80	−2.41 (−4.07 to −0.77)	−0.25 (−2.04 to 1.55)	.79
12 months	54	−2.77 (−4.24 to −1.29)	43	−1.89 (−3.11 to −0.67)	−1.08 (−2.85 to 0.69)	.23	78	−2.46 (−4.24 to −0.68)	80	−1.59 (−3.51 to 0.33)	−0.84 (−3.03 to 1.34)	.45
**Waist circumference, cm**												
Average effect of intervention	78	—	80	—	−1.81 (−3.60 to −0.03)	.05	78	—	80	—	−1.38 (−3.34 to 0.58)	.17
6 months	63	−3.25 (−4.51 to −1.99)	48	−1.34 (−3.02 to 0.33)	−2.20 (−4.14 to −0.26)	.03	78	−3.06 (−4.57 to −1.56)	80	−1.00 (−3.17 to 1.18)	−2.00 (−4.24 to 0.25)	.08
12 months	54	−2.52 (−4.15 to −0.89)	43	−2.06 (−3.70 to −0.41)	−1.08 (−3.40 to 1.23)	.36	78	−2.39 (−4.49 to −0.29)	80	−1.94 (−4.18 to 0.30)	−0.38 (−2.95 to 2.19)	.77

Abbreviations: BMI, body mass index; CI, confidence interval; DPP, Diabetes Prevention Program; VLM, virtual lifestyle management.

a Adjusted for baseline values and randomization variables: age (<50 y vs ≥50 y), BMI (<30 vs ≥30 kg/m^2^), sex (male vs female), race/ethnicity (white, African American, Hispanic, Asian and multiracial/other), and job type (office vs not office).

Mean total calories, dietary fat intakes, and saturated fat intakes decreased significantly in both intervention conditions at 6 and 12 months; however, no significant condition differences were observed (Table 3). Neither the YMCA-DPP nor the VLM-DPP yielded significant changes in moderate or vigorous physical activity or total physical activity, and no significant condition differences were observed (Table 3).

**Table 3:** Mean Changes in Diet and Physical Activity From Baseline to 6-Month and 12-Month Follow-Up With Mean Differences Between Intervention Conditions, Among Participants Who Completed Questionnaires, The City and County of San Francisco Diabetes Prevention Program Trial, 2015–2016

Variable	YMCA-DPP		VLM-DPP		Adjusted^a^ Mean Condition Difference (95% CI)	*P *Value
	N	Mean (95% CI)	N	Mean (95% CI)		
**Food energy, kcals**						
Average effect of intervention					6.90 (−134.7 to 148.47)	.92
6 months	63	−157.25 (−274.19 to −40.32)	50	−212.24 (−364.75 to −59.73)	9.64 (−137.2 to 156.49)	.90
12 months	58	−164.61 (−321.27 to −7.95)	47	−213.90 (−347.47 to −8.33)	1.37 (−167.8 to 170.51)	.99
**Fat, g**						
Average effect of intervention					−0.26 (−6.81 to 6.28)	.94
6 months	63	−8.06 (−13.46 to −2.65)	50	−10.03 (−16.82 to −3.25)	−0.60 (−7.31 to 6.11)	.86
12 months	58	−8.21 (−15.40 to −1.02)	47	−10.57 (−16.60 to −4.55)	0.62 (−7.23 to 8.47)	.88
**Saturated fat, g**						
Average effect of intervention					0.04 (−2.25 to 2.33)	.97
6 months	63	−2.86 (−4.77 to −0.97)	50	−3.76 (−6.12 to −1.41)	−0.12 (−2.51 to 2.27)	.92
12 months	58	−3.21 (−5.71 to −0.71)	47	−4.12 (−6.18 to −2.05)	0.35 (−2.33 to 3.03)	.80
**Moderate activity, min/wk**						
Average effect of intervention					6.93 (−229.2 to 243.1)	.95
6 months	30	33.9 (−145.7 to 213.6)	26	−21.44 (−232.4 to 189.5)	−34.13 (−286.8 to 218. 6)	.79
12 months	34	145.2 (−85.1 to 375.5)	18	−28.6 (−211.4 to 154.2)	100.8 (−221.4 to 423.0)	.53
**Vigorous activity, min/wk**						
Average effect of intervention					−48.1 (−211.9 to 115.7)	.56
6 months	28	12.77 (−84.57 to 110.11)	12	−35.4 (−29.1 to 219.2)	−13.0 (−187.0 to 161.0)	.88
12 months	26	−14.62 (−121.87 to 92.64)	13	3.00 (−256.4 to 316.4)	−103.6 (−292.5 to 85.3)	.28
**Total volume of physical activity, MET min/wk**						.63
Average effect of intervention					276.3 (−858.8 to 1,411.4)	.63
6 months	62	287.6 (−499.7 to 1,074.9)	38	434.3 (−1,856.4 to 2,725.0)	−475.2 (−2,435.0 to 1,484.2)	.63
12 months	55	381.6 (−393.7 to 1,156.9)	35	−159.8 (−1,274.1 to 954.5)	488.8 (−724.2 to 1,701.8)	.43

Abbreviations: BMI, body mass index; CI, confidence interval; DPP, diabetes prevention program; MET, metabolic equivalents; VLM, virtual lifestyle management.

a Adjusted for baseline values and randomization variables: age (<50 versus ≥50 years), BMI (<30 versus ≥30 kg/m^2^), sex (male versus female), race/ethnicity (white, African American, Hispanic, Asian and multiracial/other), and job type (office versus not office).

The mean number of completed core sessions was significantly higher for the YMCA-DPP (mean = 10.2 sessions; 95% CI, 9.0–11.4 sessions) than for the VLM-DPP (mean = 5.9 sessions; 95% CI, 4.7–7.1 sessions); the adjusted mean between-condition difference was 4.2 sessions (95% CI, 2.54–5.99 sessions). Participants in the YMCA-DPP were significantly more likely than those in the VLM-DPP to complete 4 or more of 16 core sessions (88.5% vs 46.2%; adjusted RR = 1.90 [95% CI, 1.49–2.43]).

## Discussion

To our knowledge, this is among the first studies to evaluate the comparative effectiveness of 2 DPP-derived lifestyle programs recognized by the CDC DPRP and delivered by trained staff from the approved organizations. In this 2-arm, parallel, randomized controlled trial of 158 employees who were at high risk for type 2 diabetes, we found no significant differences in weight loss across 12 months of follow-up between those randomly assigned to receive an in-person lifestyle program delivered at the workplace or an online lifestyle program delivered at participants’ convenience. Although we found no significant between-condition differences, both the YMCA-DPP and the VLM-DPP yielded significant reductions in body weight and percentage change in body weight at 6 months. Body weight reductions remained significant at 12 months among participants in the YMCA-DPP but not the VLM-DPP. The YMCA-DPP also resulted in reduced waist circumference at 6 months, although the finding was not significant.

Several DPP-derived lifestyle programs implemented at the workplace have been evaluated using a randomized controlled design; however, most of these interventions were delivered by research staff. In the CCSF trial, we observed a lower percentage of weight loss (from 2.4% to 2.7% at 6 months and 1.6% to 2.5% at 12 months) than that observed in previous randomized controlled trials of lifestyle interventions in workplaces. In a trial conducted at a university worksite in Ohio, participants assigned to a DPP-derived lifestyle intervention had a mean percentage weight change from baseline to 7 months of 5.5% (22). In a trial at the Pittsburg worksite of the Bayer Corporation, 45% of participants assigned to a DPP-derived intervention lost at least 5% of their body weight (23). Still, the mean weight loss observed in this trial (ranging from 1.97 to 2.14 kg at 6 months and 1.27 to 2.04 kg at 12 months across intervention conditions) is within the ranges of weight loss observed in other DPP-derived lifestyle interventions implemented at worksites, which have shown short-term weight loss of 0.7 to 5.1 kg at 3 to 6 months and long-term weight loss of 1.43 to 4.9 kg at 7 to 12 months (9). These trials used a blood test to identify individuals with prediabetes, whereas we used a diabetes risk score. It is possible that the greater weight loss observed in the workplace DPP programs that used blood tests to identify employees at risk for type 2 diabetes was related to higher levels of motivation in participants who received a diagnosis of prediabetes based on laboratory results. Although the magnitude of weight loss observed in this trial was modest, the DPP trial demonstrated that every 1 kg of weight loss resulted in a 16% reduction in type 2 diabetes risk over 3 years (24,25).

A strength of this study was our ability to randomly assign employees and thereby reduce selection bias. Using the CDC prediabetes screener was also a strength because it allowed nonclinical staff to easily identify employees at risk for type 2 diabetes and invite them to participate. An additional strength was the inclusion of a racially and ethnically diverse population of men and women, particularly given that most trials of technology-based DPP interventions conducted to date have been among primarily white populations (26). Study limitations include the loss to follow-up; however, we used multiple imputation analyses to compensate for missing data for weight loss and waist circumference. We also observed differences in sex, education, and job type among the employees who were willing versus not willing to be randomly assigned into the study; these differences highlight the need for further research on ways to recruit adults from diverse demographic and socioeconomic backgrounds in preventive lifestyle programs.

In this real-world setting, it was challenging to ensure participant engagement in each intervention program, especially for the online program. Engagement and retention in the program were greater in the YMCA-DPP, where participants were more likely to start the program and completed more sessions than participants in the VLM-DPP. Given the importance of strong engagement to successful program outcomes (27), these results suggest that offering a program at the worksite during a convenient time for employees, such as during their lunch hour or breaks, may help increase engagement and possibly effectiveness.

Since the conclusion of this trial, the CCSF has begun offering an in-person DPP-derived lifestyle program to all employees at risk for type 2 diabetes. Given the lessons learned from this trial, city officials are prioritizing recruitment and engagement activities as well as the inclusion of settings that employ workers with nonoffice jobs. As evidenced by this study and previous studies (9,10), worksites offer an opportune setting to reach adults at risk for type 2 diabetes and to offer prevention programs, potentially benefiting employees as well as employers, and potentially reducing future health care costs. CDC has developed a list of elements that should be part of workplace health programs (28), which offers guidelines for developing and evaluating such programs to increase their effectiveness and sustainability.

In conclusion, both CDC-recognized, worksite-setting lifestyle programs (6) assessed in this intervention yielded weight loss at 6 months. The workplace presents a unique opportunity to offer DPP-derived lifestyle programs.
